# Supplementary material for: Conjunctival Extramedullary Plasmacytoma in Dogs and a Cat: Clinical Characteristics and Histopathological Findings
Source: Animals (Basel). 2022 Nov 3;12(21):3015. doi: 10.3390/ani12213015 (PMC9653729; doi:10.3390/ani12213015)
Supplement: Supplementary file 1 [file animals-12-03015-s001.zip › animals-2003670-supplementary.pdf]

Table S1. Signalment and clinical data of extramedullary plasmacytoma cases (2013–2022).

Case	Species	Breed	Age (Years)	Sex	Location of Mass	OD/OS	Clinical Features	Diameter	Previous Cytology/Biopsy	Concurrent Ophthalmic Disease	Follow up
1	Dog	English Cocker Spaniel	7	M	dorsal bulbar conj.	OS	well-defined, nodular, well-vascularized, oval, superficial, salmon-colored	5 mm	No	KCS OU Distichiasis OU	1 year
2	Dog	Pinscher	15	M	Third eyelid	OD	Smooth, pink, well-defined nodule	10 mm	No	No	2 years
3	Dog	English Cocker Spaniel	11	FS	Third eyelid	OD	brownish, bilobed, exophytic mass	5 mm	No	KCS OU, pigmentary keratitis OU	2 years
4	Dog	English Cocker Spaniel	13	MC	dorsal palpebral conj.	OS	Nodular, salmon-colored, well-defined, firm	3 mm	No	No	Lost follow-up
5	Dog	Labrador Retriever	5	FS	Third eyelid	OD	well-vascularized, salmon-colored, exophytic	12mm	No	No	6 months
6	Cat	DSH	9	FS	Third eyelid	OS	smooth, pink nodule	15 mm	Yes—cytology	no	8 months
7	Dog	Maltese	10	M	Third eyelid	OD	Salmon colored, oval, exofitic, firm	8 mm	Yes—incisional biopsy	no	6 months

Abbreviations: KCS, keratoconjunctivitis sicca; FS, female-spayed; M, male; MC, male-castrated; OD, right eye; OS, left eye; OU, both eyes.

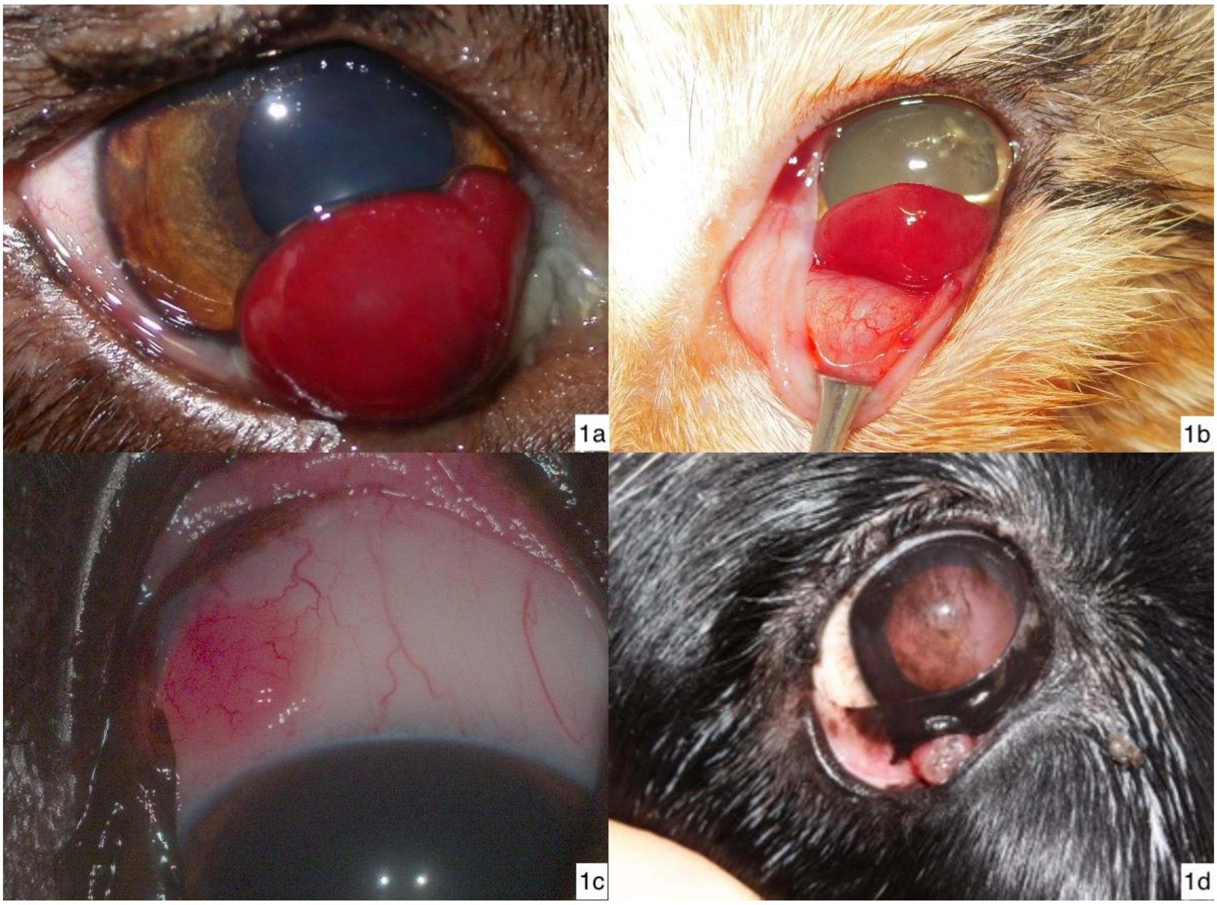

Figure S1. Gross appearance of plasmacytoma occurring on the third eyelid and the bulbar dorsal conjunctiva: (**a**) 5-year-old Labrador Retriever, (**b**) 9-year-old Domestic Short-Haired cat, (**c**) 7-year-old English Cocker Spaniel, (**d**) 11-year-old English Cocker Spaniel.

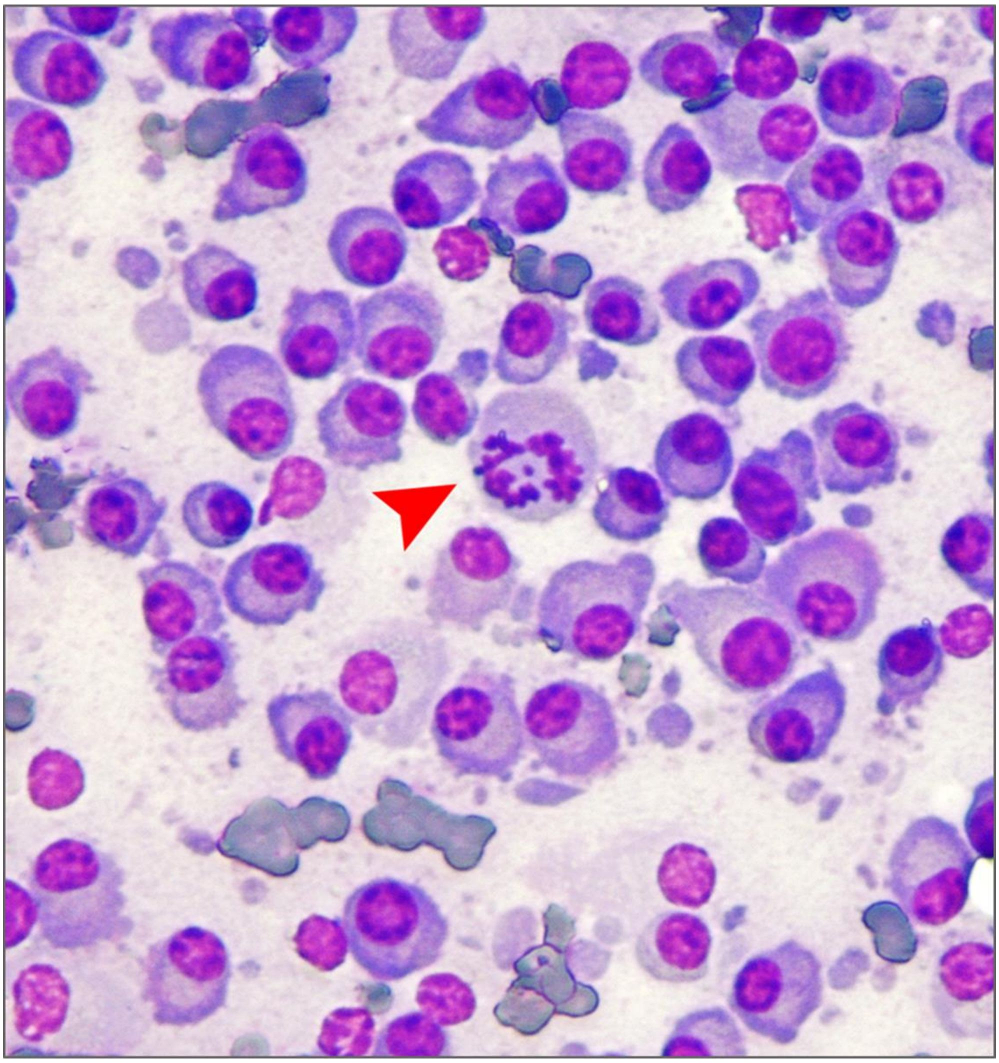

Figure S2. Case 6: cytologic image following fine needle aspiration of the mass: sheets of round cells with eccentric nucleus with cartwheel appearance, and abundant cytoplasm with clear perinuclear halo. Mitotic figures were occasionally present (arrow).

Table S2. Adjunctive diagnostic evaluations.

	Blood Work (CBC, Serum Biochemistry Profile)	Electrophoresis	Urine Analysis	Radiographic Survey	Bone Marrow Aspiration	Abdominal Ultrasound	CT
1	+	+	+	−	−	−	+
2	+	−	−	−	−	−	−
3	+	+	+	−	−	−	−
4	−	−	−	−	−	−	−
5	−	−	−	−	−	−	−
6	+	+	+	+	+	+	−
7	+	+	−	−	+	−	+

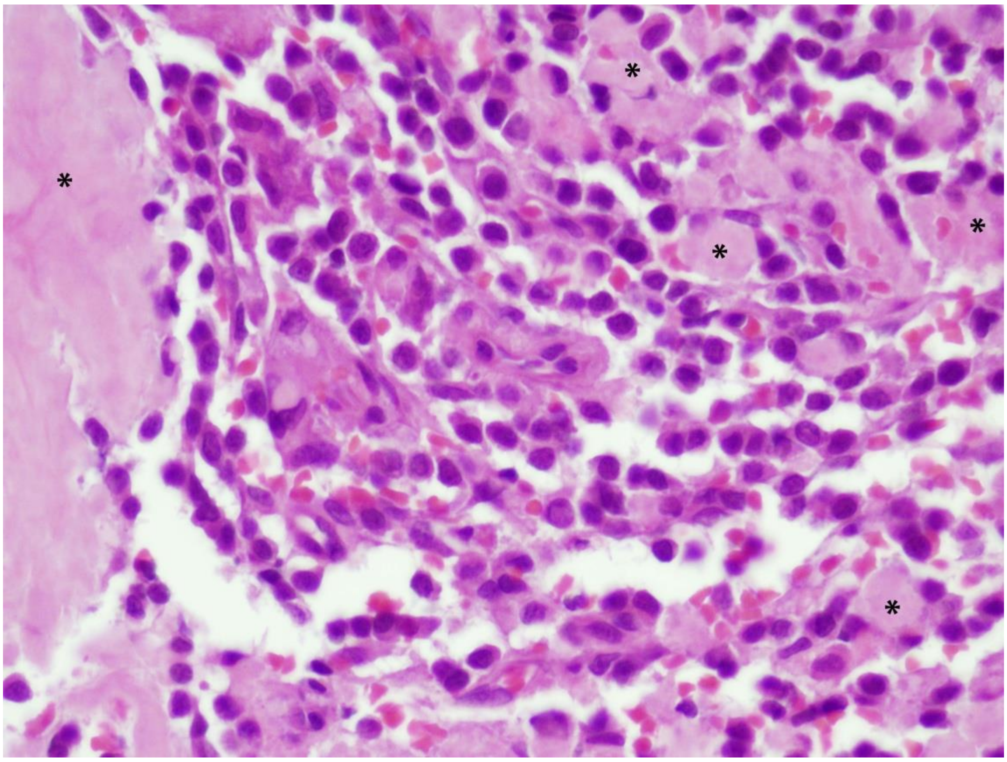

Figure S3. Case 7: Hematoxylin and eosin stain, 40 ×: the neoplastic cells were intermingled with large deposits of eosinophilic fibrillar ground substance (amyloid, asterisks).

Table S3. Immunohistochemical staining characteristics.

**Antibodies**	**Clone**	**Brand**	**Target**	**Reaction of Neoplastic Cells**
CD20	Rabbit polyclonal	Invitrogen	B lymphocytes	scattered
CD3	LN10	Novocastra	T lymphocytes	−
λ Light chain	Rabbit polyclonal	Agilent	λ Light chain (plasmacells)	+
MUM1 protein	MUM1p	Agilent	Lymphoid cells (canine plasmacytoma)	+
